# Is there sufficient *Ensifer* and *Rhizobium* species diversity in UK farmland soils to support red clover (*Trifolium pratense*), white clover (*T. repens*), lucerne (*Medicago sativa*) and black medic (*M. lupulina*)?

**DOI:** 10.1016/j.apsoil.2017.06.030

**Published:** 2017-11

**Authors:** Rachel Roberts, Robert W. Jackson, Tim H. Mauchline, Penny R. Hirsch, Liz J. Shaw, Thomas F. Döring, Hannah E. Jones

**Affiliations:** a School of Agriculture, Policy and Development, University of Reading, RG6 6AR, UK; b School of Biological Sciences, Knight Building, University of Reading, RG6 6AJ, UK; c Rothamsted Research, West Common, Harpenden, Hertfordshire, AL5 2JQ, UK; d Soil Research Centre, School of Archaeology, Geography and Environmental Science, University of Reading, RG6 6AB, UK; e Department of Agronomy and Crop Science, Faculty of Life Sciences, Humboldt Universität zu Berlin, Albrecht-Thaer-Weg 5, 14195 Berlin, Germany

**Keywords:** Legume, *Rhizobium*, *Ensifer*, Rhizobial *gyr*B, Phylogeny

## Abstract

Rhizobia play important roles in agriculture owing to their ability to fix nitrogen through a symbiosis with legumes. The specificity of rhizobia-legume associations means that underused legume species may depend on seed inoculation with their rhizobial partners. For black medic (*Medicago lupulina*) and lucerne (*Medicago sativa*) little is known about the natural prevalence of their rhizobial partner *Ensifer meliloti* in UK soils, so that the need for inoculating them is unclear. We analysed the site-dependence of rhizobial seed inoculation effects on the subsequent ability of rhizobial communities to form symbioses with four legume species (*Medicago lupulina*, *M. sativa*, *Trifolium repens* and *T. pratense*). At ten organic farms across the UK, a species-diverse legume based mixture (LBM) which included these four species was grown. The LBM seed was inoculated with a mix of commercial inocula specific for clover and lucerne. At each site, soil from the LBM treatment was compared to the soil sampled prior to the sowing of the LBM (the control). From each site and each of the two treatments, a suspension of soils was applied to seedlings of the four legume species and grown in axenic conditions for six weeks. Root nodules were counted and their rhizobia isolated. PCR and sequencing of a fragment of the *gyr*B gene from rhizobial isolates allowed identification of strains. The number of nodules on each of the four legume species was significantly increased when inoculated with soil from the LBM treatment compared to the control. Both the proportion of plants forming nodules and the number of nodules formed varied significantly by site, with sites significantly affecting the *Medicago* species but not the *Trifolium* species. These differences in nodulation were broadly reflected in plant biomass where site and treatment interacted; at some sites there was a significant advantage from inoculation with the commercial inoculum but not at others. In particular, this study has demonstrated the commercial merit of inoculation of lucerne with compatible rhizobia.

## Highlights

• Farm rhizobia communities vary; some lack lucerne and black medic-compatible *Ensifer* species.
• Red and white clover nodulated successfully but formed more nodules in the rhizobia treated soil.
• The *gyr*B sequence of the rhizobial genome differentiated between *Rhizobium* and *Ensifer* strains.
• Naturalised *Ensifer adhaerens, E. meliloti*, and *E. medicae* infected both black medic and lucerne.

## 1 Introduction

The UK is host to many agronomically important legume species in tribes Viciae (e.g. *Vicia faba*, broad bean; *Pisum sativum*, garden pea), Trifoliae (e.g. *T. repens*, white clover; *Medicago lupulina*, black medic), Lotae (e.g. *Lotus corniculatus*, birdsfoot trefoil) and Phaseolae (*Phaseolus vulgaris*, common bean). Agronomically important legumes are divided into two broad groups: large seeded ‘grain’ legumes (e.g. peas, beans), and small seeded ‘forage’ legumes which are generally sown as part of a mixed ‘ley’ with grass and used for either forage or fodder for livestock (e.g. clover, vetch). The forage legumes serve as a nitrogen supply source especially in low-input and organic farming systems, reducing dependency on mineral nitrogen, and contributing to soil fertility in the rotation.

Legumes fix nitrogen due to their symbiotic relationship with rhizobia. Here, the term ‘rhizobia’ is used to describe the 95 species of bacteria in the genus *Rhizobium* currently known to be able to form nitrogen-fixing symbioses (LPSN, 2016). These bacteria inhabit the plant root system in nodules they induce, and which provide the low oxygen environment necessary for nitrogenase to reduce atmospheric dinitrogen (N_2_) to plant usable ammonia (NH_3_). It is thought that symbiotic relationships with bacteria evolved multiple times (Martínez-Romero, 2009; Sprent, 2007). Consequently, not all rhizobia species are able to form symbiotic relationships with all legumes, and degrees of specificity exist.

At present, the most common fertility-building ley in the UK is a mix of white clover (*T. repens*) and perennial ryegrass (*Lolium perenne*). White clover is used in grazing leys owing to its high nutritional value (NIAB, 2014) and because its low apical meristem and creeping growth habit makes it tolerant to grazing. White and red clover are perennial flowering legumes naturalised in the UK, and both species have a long history of incorporation into fertility building leys, being capable of fixing between 80 and 400 kg N ha^−1^ (Taylor and Quesenberry, 1996). They are morphologically similar, but while red clover has a single crown from which multiple florets are produced (promoting an upright growth habit), white clover produces numerous stolons, or ‘runners’, promoting horizontal growth. This makes white clover more efficient at covering bare ground and tolerating grazing, but red clover more drought tolerant, owing to its single tap root (Frame et al., 1998). Clover forms a symbiotic relationship with the highly specific *Rhizobium leguminosarum sv. trifolii* (e.g. Duodu et al., 2006) which is believed to be ubiquitous in UK soils (Hirsch, 1996, Macdonald et al., 2011).

Black medic, also sometimes known as yellow trefoil (*Medicago lupulina*), is a low-growing perennial flowering legume naturalised in the UK whose long flowering period makes it a good food resource for pollinating insects (Brown, 2013). It forms a symbiotic relationship with *Ensifer medicae* (Bailly et al., 2011, Biondi et al., 2003).

Lucerne (*M. sativa*) is a member of the genus *Medicago,* and is a much less common crop in the UK but is capable of fixing large amounts of nitrogen, with estimates ranging from 100 to 500 kg N ha yr^−1^ (Anglade et al., 2015, Briggs, 2008, Marble, 1989, Nutman, 1976). Lucerne is known to form symbioses with *Ensifer meliloti* and its sister strain *E. medicae* (Bailly et al., 2011, 2006; Frame et al., 1998; Frame et al., 1998; Galibert et al., 2001; Jones et al., 2007; Mnasri et al., 2009).

In the last three decades, the three main constraints to expansion of lucerne cropping in the UK were considered to be: (1) the area of farmland meeting the necessary soil requirements, (2) the economics of growing and utilising lucerne in comparison to other forage; and (3) the economics of growing lucerne in place of arable crops (Doyle and Thomson, 1985). Now, however, there is an interest in lucerne cultivation in the UK driven by its ability to yield high quality forage under low rainfall conditions (ADHB, 2014). The resilience of lucerne in drought particularly, with the consequent change in crop gross margins, results in a major need to address the difficulties in successfully establishing this crop (DairyCo, 2014).

An interesting conundrum exists within the *Medicago*-*Ensifer* symbiosis in the UK, because lucerne (*M. sativa*) is a non-native species for which inoculation with compatible strains is considered to be necessary for successful growth, but black medic (*M. lupulina*) is a common wild plant. Evidence suggests that *E. meliloti* is the preferred symbiont for lucerne and *E. medicae* for black medic (Bailly et al., 2011); however, *E. medicae* has been isolated from lucerne (Mnasri et al., 2009). If lucerne does form symbioses preferentially with *E. meliloti*, and black medic predominantly hosts *E. medicae* and consequently increases its soil numbers, it may go some way to explaining why black medic is so prolific in the UK when lucerne is so difficult to establish: while *E. medicae* may be widespread, *E. meliloti* may still largely be absent and thus unable to infect lucerne plants.

While bacterial species dispersal is broad, it seems that the environment does indeed select – but the driving forces are specific. Community composition is influenced more by local abiotic factors such as soil type, pH and land cover than other abiotic factors such as climatic or geomorphologic characteristics (Chu et al., 2010, Dequiedt et al., 2009, Fierer and Jackson, 2006, Griffiths et al., 2011).

Few studies have been performed in the UK to determine which strains of rhizobia infect *Medicago* plants. Bailly et al. (2011) isolated only *E. medicae* from the roots of black medic (*M. lupulina*), and commercial inoculum for lucerne in the UK contains *E. meliloti.* Understanding rhizobial occurrence would be useful when advising farmers on the suitability of a given legume crop for their farm site, and is vital if successful inocula are to be produced to enable non-native legume species to be grown successfully in the UK. The aim of this research was therefore to determine whether *Trifolium pratense*, *Trifolium repens*, *Medicago lupulina* and *Medicago sativa* require inoculation for successful establishment. These species were selected based on their desirable characteristics for fertility building leys identified by Döring et al. (2013). Commercial guidelines suggest *Trifolium spp*. do not require inoculation because the symbiont *R. leguminosarum* sv. *trifolii* is ubiquitous in UK soils. In contrast, it is stated that *Medicago spp*. need to be inoculated with their symbiont *E. meliloti*.

This paper demonstrates that (i) farm rhizobia communities all possessed suitable symbionts to *Trifolium spp*., but a number of the farm sites studied lacked compatible rhizobia for the *Medicago spp*.; (ii) all legume species formed more root nodules after treatment with commercial inoculants; and (iii) *Ensifer adhaerens, E. meliloti* and *E. medicae* all infected the roots of both *Medicago spp*.; and (iv) The *gyr*B sequence of the rhizobial genome successfully differentiated between *Rhizobium* and *Ensifer* strains in this work.

## 2 Materials and methods

### 2.1 Selection of sites for use in this study

Ten sites were selected through principle component analysis (Supporting information, Fig. S1) of 34 participatory farm sites in England, Scotland and Wales as part of the LegumeLINK project conducted from 2008 to 2011 (Döring et al., 2013) by assessing variation in soil properties and choosing those sites which most differed from one another in order to select a subsample that represented the diversity of UK soils (Table 1). Each site grew 0.5 ha of a species-diverse legume based mixture (LBM) composed of 10 legume species and 4 grass species alongside a minimum of 0.5 ha of the farmer standard ley, which will not be further considered in this study. The sites were coded as CRAI and MATH (located in Scotland), SHDV and YATE (located in central England), DCHY, NWYK and TRBN (located in south west England), HDRA (located in north England), WMPS and WKNS (located in east England) (Table 2).

**Table 1:** Soil physical characteristics for all farm sites with available P, K and Mg nutrient index, and organic matter (OM) content.

Site	pH	Soil Texture	Sand%	Silt%	Clay%	Index P	Index K	Index Mg	P mg/l	K mg/l	Mg mg/l	OM%
CRAI	5.8	Sandy Loam	58	29	13	5	2-	3	94.2	179	171	7.8
DCHY	7.5	Clay	31	24	45	4	3	2	68.2	330	87	4.5
HDRA	6.6	Clay	42	21	37	3	3	3	30.4	336	108	NR
MATH	6.2	Clay Loam	43	38	19	3	2+	2	30.4	212	90	9.2
NWYK	6.1	Clay Loam	50	21	29	3	2-	2	43.6	158	84	4.2
SHDV	8	Clay Loam	32	42	26	2	1	1	21	110	35	8.2
TRBN	6.3	Clay	19	37	44	2	2+	2	22	180	60	5.5
WKNS	7.4	Clay	22	20	58	3	2-	2	31.6	122	58	NR
WMPS	8.2	Clay Loam	43	22	35	2	3	2	16.8	247	61	NR
YATE	7.1	Silty Loam	16	61	23	2	1	2	20.4	95	53	2.6

**Table 2:** Site coordinates, elevation above sea level (m) and sowing date of the LBM.

Sample Code	Site coordinates	elevation (m)
CRAI	N57:11:06 W2:12:45	109
RSWN	N50:13:24 W5:18:05	42
HDRA	N52:22:02 W1:24:47	73
MATH	N57:18:38 W2:18:29	194
NWYK	N50:46:10 W3:54:05	172
SHDV	N51:32:05 W1:29:05	162
TRBN	N50:34:12 W4:27:10	144
WKNS	N52:21:24 E1:21:08	51
WMPS	N52:08:28 W0:02:59	45
YATE	N51:26:28 W1:54:06	164

### 2.2 Inoculation of plants at experimental sites

Immediately prior to sowing the field trials, the seeds of the LBM were inoculated with three commercial inoculant types. These were specific for ‘clover’, ‘vetch’ and ‘lucerne’ and were obtained in a peat-based formulation from Legume Technology Ltd. (Eastbridgford, UK), during seed drilling at a rate of 10 g kg^−1^. Inoculants were kept cool (around 4 °C) prior to application to the seed.

### 2.3 Collection of soil samples from experimental sites

Prior to sowing, soil samples were collected from each of the sites to represent a ‘baseline’ bacterial community in either the spring or early autumn of 2008 depending upon whether the LBM was spring or autumn drilled. These uninoculated soil samples were used in this study alongside samples collected around three years later from the same sites. These later samples were taken after incorporation of the ley into the soil through tillage, and represent the state three years following the application of the inoculant to the sown seeds. Soil was taken from the upper 15 cm of the soil profile at 20 sampling points across the 0.5 ha plot at each site in a ‘W’ pattern, and stored in zip-lock bags at 4 °C until use. The plots from which soil was taken in 2009 were not known to have previously received any commercial inoculum and are referred to as the ‘control’ treatment in this study. The soil taken in 2011 had previously grown the LBM and received commercial inoculum as part of the field trials. These soil samples are referred to as the LBM treatment in this study (Supporting information Table S1).

### 2.4 Re-inoculation trials

Four legume species were selected for main trials involving the inoculation of legumes with soil suspensions from each of the ten selected sites, these were: red and white clover (which form a symbiosis with *Rhizobium leguminosarum* sv *trifolii*), and black medic and lucerne (which form symbiosis with *Ensifer meliloti*). As the symbiotic rhizobia for these four legume species were contained in the commercial inocula used in the field trials, this gave the opportunity to compare rhizobial populations in farm soils before (in 2009) and after addition (in 2011) of the inoculum. In order to test that the strain of rhizobia used for inoculation was the same as that later isolated from root nodules, each of the four plant species was also grown and inoculated with either the commercial inocula for ‘clover' and ‘lucerne' used in the original field trial (Legume Technology Ltd. Eastbridgford, UK) or with lab reference strains RCR221 and RCR2011 (Supporting information Table S3).

The growing media for inoculated plants was autoclaved fine vermiculite (1–3 mm, Sinclair, Gainsborough, UK), and involved placing 6 g into 50 ml Falcon tubes (Greiner Bio-one Ltd., Stonehouse, UK) with 20 ml sterile N-free nutrient solution containing 1 g CaPO_4_, 0.2 g K_2_HPO_4_, 0.2 g MgSO_4_·7H_2_O, 0.2 g NaCl and 0.1 g FeCl_3_ in 1 l water. Seeds were sterilised in 5 ml Hypochlorous acid HOCl solution (Hypotech Ltd., Isle of Wight, UK) diluted to 600 ppm for 7 min (small seeds, <2 mm) or 15 min (large seeds, >2 mm) and rinsed 3 times in sterilised nanopure water. Several sterilised seeds were aseptically transferred to the tubes, which were then placed in a sealed ziplock bag with a cotton wool bung to allow gas exchange. After one week, excess seedlings were removed, leaving one seedling per tube. Each seedling was inoculated with 1 ml of bacterial suspension. For experimental replicates, a soil suspension was made by mixing 10 g soil with 90 ml water, mixing for 2 min with a magnetic stirrer (Fisher Scientific UK Ltd., Loughborough, UK) at 300 rpm and adding 1 ml suspension per seedling using a wide-bore pipette. For positive controls 1 ml of overnight culture (OD_600_ = 1) was spun down in an Eppendorf Centrifuge 5415D (Eppendorf UK Ltd., Stevenage, UK) for 5 min at 5000 rpm, the supernatant removed, and cells re-suspended in 1 ml PBS. PBS was prepared using pre-made tablets (Oxoid) dissolved in sterile distilled water. For negative controls 1 ml sterile PBS was added in place of a bacterial culture.

In total 960 plants were grown, of which 720 were samples, and 240 negative controls. After 6 weeks of growth there was a mortality rate of 4.5%, thus 917 plants were harvested, comprising 685 samples and 232 negative controls. After 6 weeks, plants were harvested. Plant height and above-ground biomass were measured. Root systems were washed in water to remove vermiculite, weighed, and nodules counted. The uppermost live root nodule was excised from the root system, and placed individually in a multi-well plate. Each nodule was sterilised in 70% ethanol for 2 min, and rinsed three times in Nanopure™-purified water (Thermo Fisher Scientific Biosciences GMBH, Altrincham, UK). Nodules were then crushed in 100 μl 40% glycerol using a hedgehog replica plater, and 20 μl of the resulting cell suspension streaked onto YME agar (Per litre: Yeast extract, 1 g; Mannitol, 10 g; K_2_HPO_4,_ 0.5 g; MgSO_4_, 0.2 g; NaCl, 0.1 g; Congo red dye, 0.025 g; Agar, 15 g; Ultra-pure water, 1 l) and grown for 48–72 h at 27 °C. Rhizobial colonies were identified by the non-uptake of the Congo red dye, secretion of extra-cellular polysaccharides (resulting in readily identifiable mucoid colonies) and non-fluorescence under UV light.

When uniform colony types were obtained, a single colony was aseptically streak plated onto tryptone yeast (TY) agar (Per litre: Tryptone, 5 g; Yeast extract, 3 g; CaCl_2_·2H_2_O, 0.89 g; Agar, 17 g; Ultra-pure water,1 l) to reduce inhibitory polysaccharide production; this ensured strain purification. Overnight cultures were made by aseptically transferring a single colony with a loop into 10 ml TY broth (recipe as before) and incubating at 27 °C on a rotary shaker for 24–72 h at 200 rpm, until samples became turbid. Frozen stocks were made by combining 700 μl of overnight culture with 300 μl 40% glycerol in a cryovial. These were then stored at −80 °C until required.

### 2.5 Collection and isolation of bacterial strains

All strains used in this study were either isolated from the root systems of legumes inoculated with soil from the participatory farm sites or accessed from the Rothamsted culture collection (Rothamsted Research, Harpenden, UK) (Supporting information Table S3). The Rothamsted culture strains were: *Rhizobium leguminosarum* sv *trifolii* (strain no: RCR221, RCR226), *Rhizobium leguminosarum* sv *viciae* (strain no: RCR1001), *Ensifer meliloti* (strain no: RCR2011), *Rhizobium gallicum* sv *gallicum* (strain no: RCR3007), and *Mesorhizobium loti* (strain no: RCR3002, RCR3209).

### 2.6 DNA extraction

Frozen stocks of overnight cultures of rhizobia grown in TY broth, were used for DNA extractions with the Puregene Yeast/Bacteria Kit B (Qiagen Sciences, Maryland, USA) following the manufacturer’s protocol for Gram-negative bacteria. DNA concentration was measured using a NanoDrop Spectrophotometer ND-1000 (Labtech International Ltd., Uckfield, UK) and ND-1000 v 3.8.1software.

### 2.7 Amplification of *gyr*B gene DNA fragments by PCR

Bacterial DNA was amplified using *gyr*B specific primers (gyrBf3-F: ATGTGGTGGAACGAYAGCTA and gyrBr5-R: TCCTGGATRAAKTCGCG (Mauchline et al., 2014)), which amplified a fragment of the *gyr*B gene, and are specific to rhizobia. PCR was conducted using *Taq* DNA polymerase (Bioline), PCR buffer ((NH_4_)_2_SO_4_) (10×) and 50 mM MgCl_2_ from Bioline (London, UK); each dNTP (Fermentas Life Sciences, Burlington, Canada) at a concentration of 200 μM. Large-scale PCRs using *gyr*B primers were conducted using a PTC-100 Programmable Thermal Controller (MJ Research, Inc.). PCR amplification programmed at 1 cycle at 94 °C for 3 min, 30 cycles at 94 °C for 1 min, at 60 °C for 1 min, and at 72 °C for 2 min; with a final extension at 72 °C for 3 min. PCR products were separated on a 1% agarose gel in TAE buffer. Purification of PCR products was conducted by Source Bioscience Ltd. (Cambridge, UK). Sequencing was conducted by Source Bioscience Ltd. (Cambridge, UK).

### 2.8 Sequence analysis of gene fragments

Raw sequence data was analysed in Geneious v 6.1 (Biomatters, Auckland, New Zealand). Incomplete (i.e. <500 bp) and low resolution sequences were removed. A MAFFT alignment was conducted and trace data analysed to correct mis-attributed nucleotides. A ‘trimmed’ section of 573 bp in length was used for further analysis of potential single nucleotide polymorphisms (SNPs).

Phylogenetic trees were constructed in Geneious using the PHYML builder and Hasegawa-Kishino-Yano substitution model, with bootstrapping set at 100 replicates. Design elements of phylogenetic trees were edited using Dendroscope v 3.0 (Huson and Scornavacca, 2012). Sequences were then compared against the BLAST nucleotide database to identify rhizobia species names. Additional *gyr*B sequences from *R. leguminosarum* sv. *trifolii* strains isolated from Africa, South America and Australia were used for comparison during analysis (Mauchline et al., 2014).

### 2.9 Data analysis

For selection of sites, a principal component analysis was conducted using soil data relating to pH, texture and mineral composition obtained from the LegumeLINK project (Döring et al., 2013). Five clusters were identified and a site chosen from each one; when supplemented with five outlying data points this resulted in the selection of the most diverse and representative selection of sites (Supporting information Fig. S1). Together, principal components 1 and 2 (PC1 and PC2) accounted for 92% of the variation in the dataset. Latent vectors indicated that sites that had low PC1 scores were linked to high sand content, and low PC2 scores to high soil P content. Sites GLDG, PHFM and RHYD were not used owing to a lack of legume species data available.

Data for the number of nodules per plant was transformed using the square root function and analysed using REML (Genstat 17th edition) with the fixed model: treatment + plant + treatment × plant and the random model: site + site × replicate. All other data was analysed with the programme R, v. 3.0.0 (R Core Team, 2013). Shoot weight, root weight and total weight were analysed with treatment and site as fixed factors. In the majority of cases non-transformed data showed normally distributed residuals; in some cases log-transformation was necessary to normalise residuals. Only in one case (black medic shoot weight) there was no possibility of achieving normality of residuals through transformation, however significance levels in this case were robust against any transformation. Nodule presence (i.e. the proportion of plants with vs. without nodules) was analysed with GLM procedure using quasibinomial error structure in case of over dispersion.

## 3 Results

The soil at the 10 selected farm sites spanned the pH range of 5.8–8.2, ranging from sandy loam to clay, with the breadth of P, K and Mg nutrient index values (Table 1).

### 3.1 Nodulation of legume species

Of the 685 sample plants, a total of 464 formed nodules. A small number of lucerne plants formed ‘pseudo’ nodules: growths on the roots resembling nodules, but which are white in colour and do not contain rhizobia. These nodules were not counted in the analysis as they did not contain rhizobia; none of the 232 negative controls formed nodules. When grown in the absence of compatible rhizobia, plants showed both stunted growth and chlorosis of leaves; this was true for all non-nodulated samples, whether negative control or a plant which received a soil suspension but failed to form nodules. The treatment, whether the plants were grown in the control farm soil, or in soil where the inoculated LBM had grown, had an effect on the number of nodules present on black medic and lucerne. Across all sites the mean percentage nodulation was 26% for black medic and for lucerne, this increased to a mean of 49% and 45%, respectively, in soil from the LBM treatment. This treatment effect was not evident for red or for white clover where the level of nodulating plants did not significantly diverge from a 100% nodulation success (Table 3). However, when considering the number of nodules per plant, the LBM treatment had a favourable effect on the number of nodules each species formed compared to that of control plots for all species (*P <* 0.001). The greatest increase in the number of nodules per plant was recorded for red clover from a mean of 5.45 per plant to 9.35 per plant (Table 4).

**Table 3:** Total number of plants forming root nodules for each plant species and treatment by site. Black medic (BM), lucerne (LU), red clover (RC) and white clover (WC). Control: from sites not treated with commercial inoculum, LBM: from sites previously treated with commercial inoculum.

	BM		LU		RC		WC	
Site	LBM	Control	LBM	Control	LBM	Control	LBM	Control
	Plants nodulated	Plants nodulated	Plants nodulated	Plants nodulated	Plants nodulated	Plants nodulated	Plants nodulated	Plants nodulated
CRAI	0 (0%)	0 (0%)	2 (22%)	0 (0%)	9 (100%)	9 (100%)	9 (100%)	8 (100%)
DCHY	8 (100%)	8 (89%)	8 (100%)	9 (100%)	9 (100%)	9 (100%)	9 (100%)	9 (100%)
HDRA	8 (89%)	0 (0%)	7 (78%)	3 (38%)	8 (100%)	8 (100%)	9 (100%)	9 (100%)
MATH	0 (0%)	0 (0%)	1 (14%)	0 (0%)	9 (100%)	9 (100%)	9 (100%)	9 (100%)
NWYK	0 (0%)	0 (0%)	0 (0%)	0 (0%)	6 (75%)	9 (100%)	7 (100%)	9 (100%)
SHDV	1 (11%)	7 (88%)	0 (0%)	4 (50%)	8 (100%)	7 (100%)	8 (100%)	9 (100%)
TRBN	1 (11%)	0 (0%)	4 (44%)	0 (0%)	9 (100%)	9 (100%)	9 (100%)	9 (100%)
WKNS	8 (100%)	7 (78%)	5 (56%)	4 (44%)	9 (100%)	9 (100%)	9 (100%)	9 (100%)
WMPS	9 (100%)	0 (0%)	9 (100%)	2 (22%)	6 (100%)	6 (67%)	9 (100%)	5 (100%)
YATE	8 (89%)	0 (0%)	3 (38%)	0 (0%)	9 (100%)	9 (100%)	9 (100%)	9 (100%)
TOTAL	43 (49%)	22 (26%)	39 (45%)	22 (26%)	82 (98%)	84 (97%)	87 (100%)	85 (100%)

**Table 4:** Mean number of root nodules formed for each plant species and treatment by site. Black medic (BM), lucerne (LU), red clover (RC) and white clover (WC). Control: from sites not treated with commercial inoculum, LBM: from sites previously treated with commercial inoculum.

	BM		LU		RC		WC	
Site	LBM	Control	LBM	Control	LBM	Control	LBM	Control
	Mean no. nodules ± 1 SE	Mean no. nodules ± 1 SE	Mean no. nodules ± 1 SE	Mean no. nodules ± 1 SE	Mean no. nodules ± 1 SE	Mean no. nodules ± 1 SE	Mean no. nodules ± 1 SE	Mean no. nodules ± 1 SE
CRAI	0 ± 0	0 ± 0	3.0 ± 2.0	0 ± 0	10.1 ± 1.7	5.2 ± 1.2	5.6 ± 0.9	4.8 ± 0.8
DCHY	4.4 ± 1.0	2.6 ± 0.3	3.6 ± 0.8	3.3 ± 0.6	13.0 ± 2.5	4.7 ± 0.8	6.8 ± 1.5	2.6 ± 0.4
HDRA	4.3 ± 0.8	0 ± 0	5.3 ± 1.7	1.0 ± 0	11.9 ± 1.5	7.0 ± 0.5	5.2 ± 1.3	7.7 ± 0.6
MATH	0 ± 0	0 ± 0	0 ± 0	0 ± 0	8.8 ± 1.9	4.2 ± 0.8	6.7 ± 1.4	2.1 ± 0.4
NWYK	0 ± 0	0 ± 0	0 ± 0	0 ± 0	3.3 ± 0.6	1.0 ± 0.6	3.6 ± 0.5	3.3 ± 0.3
SHDV	1.0 ± 0	3.7 ± 1.1	0 ± 0	1.8 ± 0.3	10.9 ± 1.9	6.6 ± 0.9	5.1 ± 0.7	4.9 ± 1.0
TRBN	2.0 ± 0	0 ± 0	1.8 ± 0.8	0 ± 0	10.0 ± 2.3	5.3 ± 0.5	6.9 ± 0.6	7.3 ± 1.1
WKNS	3.8 ± 0.6	2.4 ± 0.6	10.8 ± 4.3	1.5 ± 0.5	9.4 ± 1.3	8.2 ± 1.2	6.7 ± 0.6	5.6 ± 0.5
WMPS	3.3 ± 0.6	0 ± 0	1.8 ± 0.4	1.5 ± 0.5	7.8 ± 1.9	2.3 ± 0.6	4.6 ± 0.6	2.0 ± 0.6
YATE	2.0 ± 0.6	0 ± 0	1.0 ± 0	0 ± 0	8.6 ± 1.0	9.0 ± 1.5	6.1 ± 0.5	5.8 ± 1.2
TOTAL	3.4 ± 0.33	2.9 ± 0.41	3.9 ± 0.78	2.2 ± 0.33	9.6 ± 0.60	5.6 ± 0.36	5.8 ± 0.30	4.8 ± 0.31

When all species are considered together there are significant site by treatment effects for nodule presence in the LBM compared to the control soils (*P* < 0.001). The effect is principally the result of the variability observed for black medic and lucerne between sites. Sites can be generally grouped in to those which (i) supported high nodulation of all species in the control and the LBM soil: DCHY, WKNS; (ii) at which lucerne and black medic did not thrive irrespective of treatment: CRAI, MATH, NWYK; and (iii) the LBM treatment had a positive effect on nodulation for BM and LU: HDRA, SHDV, WMPS, YATE. The grouping of TRBN could be considered intermediate. Group one included species with soil pH 5.3 and 7.0; group 2 soil pH ranging from 4.9 to 6.0; and group 3 pH ranging from 5.5 to 7.3 (Table 1). A larger data set is required for analysis of individual species for site by treatment effects, although based on the existing data set there is no clear evidence that pH, soil phosphorus, potassium or soil carbon individually had an effect on nodulation success.

### 3.2 Biomass of legume plants

The failure of a plant to form nodules resulted in chlorosis and stunted growth. This resulted in a significantly lower mean total plant weight for non-nodulated compared to nodulated plants. The LBM treatment had a positive effect on the aboveground biomass of red (*P* = 0.003) and white clover (*P <* 0.001) (Table 5). Site had a significant effect on the biomass of all plant species (*P <* 0.001) with a significant interaction between site and treatment (*P <* 0.001). Below ground, root biomass increased in the LBM treatment compared to the control for BM (*P* = 0.01), and for LU (*P* = 0.002) there was a tendency for this to be the case for the red clover (*P* = 0.085) but not white clover (Table 5). The total biomass of plants demonstrated significant treatment effects for LU (*P* = 0.002), RC and WC (*P <* 0.001). Effect of treatment was not detected in black medic due to highly significant interaction effects between site and treatment which were also detected for all other species (*P <* 0.001). For all species, site had an effect on total plant biomass which was greater for LU, WC and RC (*P <* 0.001) than for BM (*P* = 0.02). When all species were considered together, there were highly significant site × treatment effect. This is mainly due to BM and LU and is caused by some sites showing strong differences between LBM and control (HDRA), while others did not show similar effects, or even had reversed effects (SHDV). When species are considered singly, the model is over specified and site × treatment effects cannot be tested.

**Table 5:** Above ground (shoot) and below ground (root) biomass (g) for black medic, lucerne, red clover and white clover after 6 weeks of growth in non-inoculated (control) and LBM treated soils across the 10 farm sites. The mean values given are for 3 replicate plants, and the standard deviation is given for each treatment and site combination for the 4 plant species.

	Black medic				Lucerne				Red clover				White clover			
	LBM		control		LBM		control		LBM		control		LBM		control	
site	Mean	SD	Mean	SD	Mean	SD	Mean	SD	Mean	SD	Mean	SD	Mean	SD	Mean	SD
Shoot																
CRAI	0.0234	0.0065	0.0699	0.0718	0.0407	0.0147	0.0285	0.0053	0.2647	0.0488	0.2196	0.0701	0.3215	0.0597	0.2761	0.0686
DCHY	0.1425	0.0228	0.0380	0.0055	0.1799	0.0589	0.0691	0.0017	0.2248	0.0709	0.0812	0.0156	0.1847	0.1241	0.0315	0.0061
HDRA	0.0620	0.0452	0.0336	0.0080	0.0641	0.0494	0.0393	0.0095	0.2529	0.0287	0.3165	0.0851	0.2482	0.0916	0.3344	0.0867
MATH	0.0251	0.0031	0.0294	0.0081	0.0396	0.0055	0.0411	0.0124	0.1241	0.0171	0.0647	0.0129	0.2578	0.0969	0.0252	0.0048
NWYK	0.0315	0.0181	0.0338	0.0114	0.0409	0.0086	0.0436	0.0080	0.1219	0.0359	0.1836	0.0537	0.1825	0.0550	0.1541	0.0153
SHDV	0.0277	0.0064	0.1314	0.0247	0.0276	0.0051	0.0491	0.0061	0.1970	0.0550	0.1802	0.0026	0.1143	0.0965	0.1461	0.0300
TRBN	0.0649	0.0701	0.0282	0.0048	0.1262	0.0778	0.0493	0.0131	0.2521	0.1114	0.1789	0.0421	0.3661	0.0859	0.1512	0.0156
WKNS	0.1013	0.0251	0.0893	0.0150	0.0682	0.0294	0.0836	0.0789	0.1655	0.0071	0.1814	0.0257	0.1647	0.0324	0.1841	0.0143
WMPS	0.1629	0.0368	0.0909	0.0290	0.2235	0.0122	0.1485	0.0195	0.3401	0.0602	0.1373	0.0526	0.4074	0.0362	0.1156	0.0117
YATE	0.0289	0.0030	0.1094	0.0134	0.0863	0.0506	0.2091	0.0467	0.1461	0.0139	0.1295	0.0171	0.1568	0.0149	0.0840	0.0105

Root																
CRAI	0.0611	0.0221	0.1353	0.0240	0.2160	0.0446	0.1521	0.0425	0.1178	0.0324	0.1750	0.0388	0.1809	0.0374	0.1224	0.0192
DCHY	0.0995	0.0195	0.1998	0.0572	0.2183	0.0903	0.0918	0.0450	0.0328	0.0092	0.0653	0.0349	0.0933	0.0743	0.0217	0.0021
HDRA	0.1062	0.0299	0.1827	0.0115	0.2044	0.0509	0.0956	0.0545	0.1023	0.0219	0.2265	0.0241	0.1960	0.0226	0.1615	0.0315
MATH	0.0751	0.0240	0.0922	0.0063	0.0724	0.0261	0.0937	0.0317	0.0521	0.0091	0.0918	0.0205	0.0449	0.0026	0.0315	0.0278
NWYK	0.1011	0.0374	0.1556	0.0578	0.1355	0.0045	0.0824	0.0239	0.0866	0.0335	0.1644	0.0150	0.1352	0.0047	0.0764	0.0185
SHDV	0.0752	0.0251	0.1293	0.0372	0.1769	0.0404	0.0533	0.0308	0.1094	0.0374	0.1470	0.0514	0.1977	0.0839	0.0692	0.0095
TRBN	0.0891	0.0331	0.2225	0.1006	0.1854	0.0367	0.1242	0.0122	0.0699	0.0200	0.1408	0.0434	0.1326	0.0416	0.0639	0.0078
WKNS	0.0649	0.0254	0.0894	0.0153	0.0953	0.0101	0.0877	0.0574	0.0719	0.0103	0.0902	0.0170	0.0863	0.0026	0.0623	0.0191
WMPS	0.0887	0.0161	0.1957	0.0253	0.1881	0.0480	0.0989	0.0141	0.0307	0.0098	0.0818	0.0738	0.1364	0.0401	0.1626	0.0418
YATE	0.1127	0.0218	0.2325	0.0948	0.1304	0.0370	0.0598	0.0161	0.0387	0.0008	0.0460	0.0060	0.2153	0.0367	0.1558	0.0179

### 3.3 Analysis of *gyr*B gene fragments from red and white clover strains

The sequences of 191 rhizobial strains isolated from red and white clover plants held a high consensus (>98%) with the sequenced genome of *R. leguminosarum* sv *trifolii* WSM1325 (accession CP001622) (Reeve et al., 2010b), with which strain RCR221 and the commercial inoculum were found to show high consensus. This strain was found at all sample sites, and in both red and white clover.

**Fig. 1:**
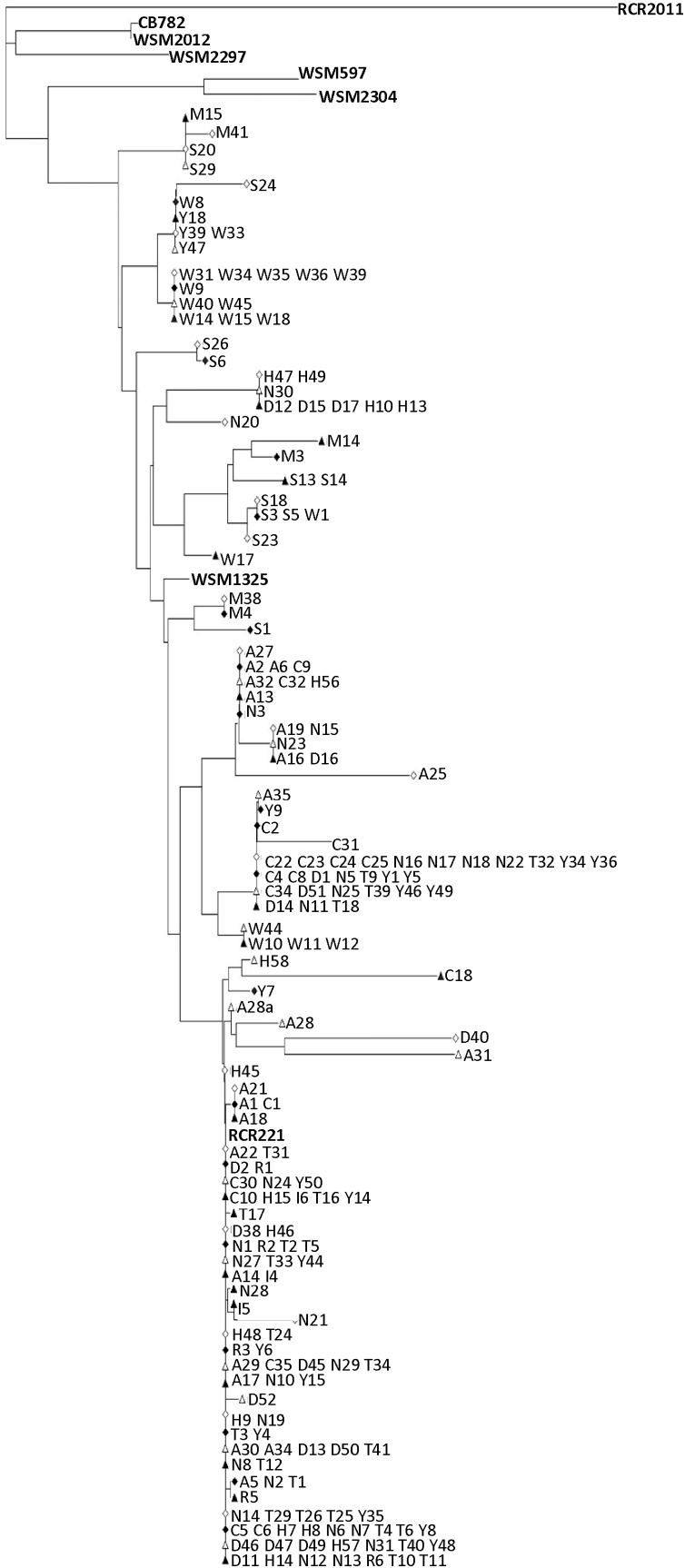
Phylogenetic tree of *Rhizobium leguminosarum* strains isolated from white and red clover root systems inoculated with soil from each of 10 trial sites. Rooted using *Ensifer meliloti* reference strain RCR2011. Alpha-numeric code refers to bacterial strain (Supporting information Table), with letters representing different sites. Reference strains are shown in bold; strains isolated from plants treated with commercial Inoculant or Reference strains shown in italics. ◊/♦: white clover △/▲: Red clover; ○/●: Black medic; □/■: Lucerne. Empty node icon: site not previously inoculated with strain RCR2011; filled node icon: site previously inoculated with strain RCR2011.

No clear difference can be seen in groupings for strains isolated from red and white clover, or between sites. Strain WSM1325 is the parent node for the majority of isolated strains, indicating that it represents a clade that is ubiquitous. As expected, strains recovered from control plants held high consensus with strain RCR221 (3), the commercial inoculation strain. A fragment of the protein encoding and essential housekeeping topoisomerase gene, DNA gyrase subunit B (*gyrB*), was used for phylogenetic analysis, enabling a finer resolution of isolates than possible by the 16S rRNA gene (Mauchline et al., 2014). Although no difference was found between strains at a local level, when a new alignment was conducted incorporating *gyr*B sequences for strains isolated from outside Europe, two new clades were formed at the root of the tree: one with strains originating from Africa, and the other South America, in agreement with the findings of Mauchline et al. (2014). They were identified as *R. leguminosarum* sv. *trifolii* strains CB782 and WSM2304 (Table 6).

**Table 6:** Identity of overseas rhizobial strains used in alignment with UK rhizobia strains isolated in this experiment.

Strain	Continent of origin	Identity of strain
AfrP-SA-CB782	Africa	*R. leguminosarum* sv. *trifolii* CB782
AfrP-SA-WSM2297		
AfrP-Eth-WSM2012		
SAP-Uru-WSM2304	South America	*R. leguminosarum* sv. *trifolii* WSM2304
EurA-Uru-WSM597		
This study	Europe	*R. leguminosarum* sv. *trifolii* WSM1325

### 3.4 Analysis of *gyr*B gene fragments from lucerne and black medic strains

**Fig. 2:**
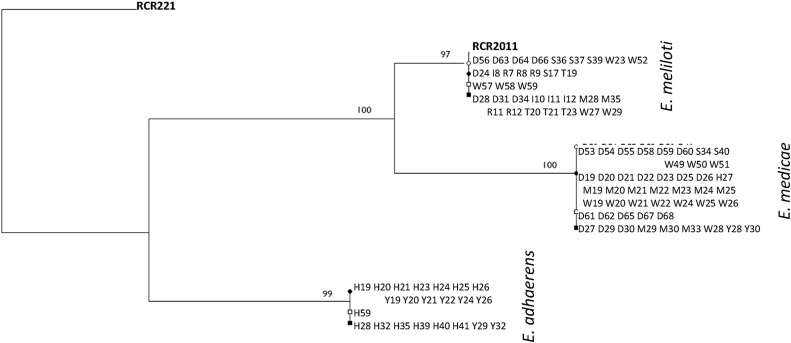
Phylogenetic tree of *Ensifer* strains isolated from black medic and lucerne root systems inoculated with soil from each of 10 trial sites. Rooted using *Rhizobium leguminosarum* sv. *trifolii* reference strain RCR221; bootstrapping values are shown on branches. Alpha-numeric code refers to bacterial strain, with letters representing different sites. Reference strains are shown in **bold**; strains isolated from plants treated with commercial Inoculant or Reference strains shown in italics. ♢/♦: white clover △/▲: Red clover; ○/●: Black medic; □/■: Lucerne. Empty node icon: site not previously inoculated with strain RCR2011; filled node icon: site previously inoculated with strain RCR2011.

In total, the *gyr*B gene fragment sequences of 103 rhizobial strains isolated from black medic and lucerne plants were analysed. They fell into 3 distinct groupings, showing high consensus with *gyr*B in the sequenced genomes of *E. meliloti* RCR2011 (accession CP004140) (Sallet et al., 2013), *E. medicae* WSM419 (CP000738) (Reeve et al., 2010a) and *E. adhaerens* OV14 (CP007236) (Rudder et al., 2014) (Fig. 2).

## 4 Discussion

The majority of clover plants in this study formed nodules, supporting previous findings that *R. leguminosarum* sv *trifolii* is ubiquitous in UK soils (Hirsch, 1996, Macdonald et al., 2011) and that inoculation with rhizobia is not required for successful nodulation in clover.

While inoculation is not necessary for nodulation to take place, red and white clover both formed significantly more root nodules when inoculated with soil from the LBM treatment. The majority of black medic and lucerne plants did not form nodules in the control treatment, which either suggests that *Ensifer* strains did not survive storage of soil samples from 2008 to the time of analysis in 2012–2014, or that they were not present in the soil to begin with. *Ensifer* strains have been recorded to successfully survive storage for 35 years in dried soil and to subsequently infect lucerne plants (Jensen, 1961) therefore it is suggested that the compatible strains for black medic and lucerne were simply not present in sufficient numbers to initiate symbioses in a number of farm soils. However, the assessment of inactive nodules, particularly relevant in lucerne and black medic, compared to the total number of nodules per plant, would have provided a more complete analysis of the relative efficiency of nodulation at the individual farm sites.

Nodulation rates of both black medic and lucerne were significantly higher when inoculated with soil suspensions from LBM soil than with control soil. This suggests that the rhizobial inoculant applied at the start of the field trial in 2009, but after the sampling of control soil, had either persisted in the soil and remained able to infect legumes after 3 years or were active within lucerne and black medic in the LBM plots (Hirsch, 2010).

Nodulation rates for black medic and lucerne in the control treatment were particularly high at site DCHY. One explanation is that, unknown to us; this site may have previously received a lucerne inoculum that had persisted in the soil, despite the lack of hosts. Rhizobia are known to be competent saprophytes. The fact that the site has a mildly alkaline soil may have helped strains to survive, as rhizobia numbers have been found to decrease over time in acid soils (Fettell et al., 1997).

In all these sites which used rotation as part of standard organic management and consequently omitted the use of mineral nitrogen, *R. leguminosarum* sv. *trifolii* may be maintained at higher numbers than, for example, in conventionally-managed cereals. Following this logic, as black medic and lucerne are grown much less frequently than clover, populations of compatible rhizobia are not maintained. Indeed, Svenning (2001) found that inoculum levels of *R. leguminosarum* sv. *trifolii* remained high in soils until the clover crop was removed, and Da and Deng (2003) found that the survival of *E. meliloti* inocula was improved by lucerne cropping. If this is the case, the recommendation for farmers is clearly to grow a diverse range of species regularly to build up and maintain soil ‘stores' of compatible rhizobia, although inoculation may be required initially.

When plants were inoculated with fresh soil that had hosted a diverse legume crop for three years (and had been inoculated with rhizobia three years previously) the number of plants forming nodules, and the number of nodules was higher.

Sequencing the *gyrI* section of the rhizobial genome proved to be a useful identifier between *Rhizobium* and *Ensifer* strains, although this is one possible of gene of many, which are need to confirm the distinct identity of each accession. The *gyr*B gene of *R. leguminosarum* sv. *trifolii* revealed small variations between UK isolates. This is in agreement with previous work that revealed groupings between isolates derived from different continents (Mauchline et al., 2014).

While the *gyr*B subunit was found to be useful for SNP detection between *R. leguminosarum* sv. *trifolii* genes this did not prove to be the case for *Ensifer* strains. For examination of more subtle genomic variations, the use of another gene or technique should be investigated, for example the use of multilocus sequence typing (MLST), which compares genetic variations in several genes together and has been found successful in other bacterial species (e.g. *Pseudomonas syringae*) (Sarkar and Guttman, 2004).

All three *Ensifer* strains were found to infect both black medic and lucerne with no obvious preference contrary to previous suggestions that *E. meliloti'*s preferred symbiont is lucerne (e.g. Galibert et al., 2001). This flexibility in selection of symbiont between *Medicago* species and rhizobia highlights the increased specificity of clovers to one symbiovar of rhizobia (*R. leguminosarum* sv*. trifolii*).

All three *Ensifer* strains could be found as naturalised strains in soils. While the presence of *Ensifer* strains may be explained by the widespread growth of black medic as a wildflower, the presence of isolates with *gyr*B sequences most closely resembling *E. adhaerens* at two of the sites is surprising. The 16S rRNA sequence is required to confirm whether *E. adhaerens* has indeed been identified. If however, this species has been isolated, it was originally discovered in China, *E. adhaerens* is known primarily for its ability to effectively nodulate soybeans (*Glycine max*) (Scholla and Elkan, 1984) – a genus separated from *Medicago* by 20 million years of evolution (Choi et al., 2004). It has previously been found to nodulate lucerne (Chen et al., 1988), and to be closely related to *E. meliloti* strain 1021 (Crespo-Rivas et al., 2009). However, these UK isolates might represent a new species that groups more closely with *E. adhaerens* than with *E. meliloti* or *E. medicae.*

## 5 Conclusion

Given the interest in expanding lucerne cultivation in the UK, the finding that the UK distribution of lucerne rhizobial symbionts is not ubiquitous has current relevance for guiding inoculation strategy. This study confirms the ubiquity of *R. leguminosarum* sv. *trifolii* in UK soils, which was present at all sites, with little variation detected. *Ensifer* strains were found in some soils, assumed to be naturalised from previous inoculation, in areas spanning the East to the South-West of the UK geographical locations (Cornwall, Suffolk and Cambridgeshire). Isolates that grouped with the non-native species *Ensifer adhaerens* were only isolated from two sites, both without any history of inoculation.

The growth of legume leys will counter increasing synthetic N and protein feed costs will provide sufficient financial benefit to encourage growth of legumes such as lucerne in the absence of subsidies. Information from this study indicates that inoculation will be beneficial until sufficient numbers become naturalised.
