# Supplementary material for: Is there sufficient Ensifer and Rhizobium species diversity in UK farmland soils to support red clover (Trifolium pratense), white clover (T. repens), lucerne (Medicago sativa) and black medic (M. lupulina)?
Source: Appl Soil Ecol. 2017 Nov;120:35–43. doi: 10.1016/j.apsoil.2017.06.030 (PMC5637928; doi:10.1016/j.apsoil.2017.06.030)
Supplement: Supplementary file 1 [file mmc1.docx]

# Supplementary information

**Figure S1**: Principal component analysis (PCA) using soil data for 34 farm sites. Some sites were identified which had common characteristics; only one farm from each group was selected with exception TRBN due to the close proximity of two such groupings within one area of the plot. x axis = PC1, y axis = PC2. Underlined sites are those chosen for this study.

**
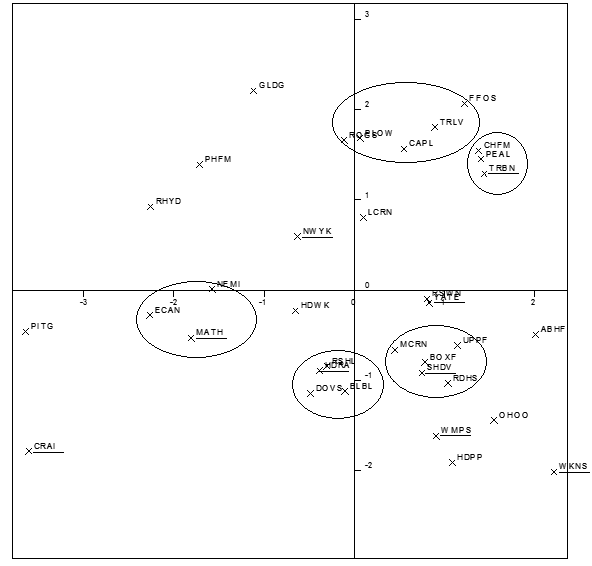
**

| **Common Name** | **Latin Name** | **Code** | **Seed Variety** | **Seed rate (g m^-2^)** | **Thousand**  **Grain**  **Weight (g)** | **Inoculum type** |
| --- | --- | --- | --- | --- | --- | --- |
| White clover | *Trifolium repens* | WC | Alice | 0.15 | 0.5 | Clover |
| Red clover | *Trifolium pratense* | RC | Merviot | 0.25 | 1.8 | Clover |
| Alsike clover | *Trifolium hybridum* | AC | Dawn | 0.125 | 0.7 | Clover |
| Crimson clover | *Trifolium incarnatum* | CC | Coutea | 0.225 | 3.1 | - |
| Black medic(k) | *Medicago lupulina* | BM | Virgo Pajberg | 0.25 | 1.6 | Lucerne |
| Sainfoin | *Onobrychis viciifolia* | SF | Esparsette | 0.5 | 19.2 | - |
| Lucerne/ alfalfa | *Medicago sativa* | LU | Daisy | 0.25 | 2.4 | Lucerne |
| Birdsfoot trefoil | *Lotus corniculatus* | BT | San Gabrielle | 0.25 | 1.2 | - |
| Large birdsfoot trefoil | *Lotus pedunculatus* | LT | Maku | 0.25 | 1 | - |
| Meadow pea | *Lathyrus pratensis* | MP | - | 0.325 | 153 | Vetch |
| Italian ryegrass | *Lolium multiflorum* | IR | Teana | 0.1 | 2.9 | - |
| Meadow fescue | *Festuca pratensis* | MF | Rossa | 0.125 | 2.14 | - |
| Perennial ryegrass | *Lolium perenne* | PR | Orion | 0.25 | 2 | - |
| Timothy | *Phelum pratense* | T | Dolina | 0.05 | 0.32 | - |

**Table S1** Composition of the All Species Mixture (LBM). Data courtesy of the LegumeLINK project.

| **Site code** | **Site treatment** | **Plant species** | **Strain reference** |
| --- | --- | --- | --- |
| CRAI | LBM | White Clover | C1-9 |
| CRAI | LBM | Red Clover | C10-18 |
| CRAI | Control | White Clover | C20-26 |
| CRAI | Control | Red Clover | C27-35 |
| DCHY | LBM | White Clover | D1-9 |
| DCHY | LBM | Red Clover | D10-18 |
| DCHY | LBM | Black Medic | D19-26 |
| DCHY | LBM | Lucerne | D27-34 |
| DCHY | Control | White Clover | D35-43 |
| DCHY | Control | Red Clover | D44-52 |
| DCHY | Control | Black Medic | D53-60 |
| DCHY | Control | Lucerne | D61-68 |
| HDRA | LBM | White Clover | H1-9 |
| HDRA | LBM | Red Clover | H10-18 |
| HDRA | LBM | Black Medic | H19-27 |
| HDRA | LBM | Lucerne | H28-35 |
| HDRA | Control | White Clover | H42-49 |
| HDRA | Control | Red Clover | H51-58 |
| HDRA | Control | Lucerne | H59-60 |
| MATH | LBM | White Clover | A1-8 |
| MATH | LBM | Red Clover | A10-18 |
| MATH | Control | White Clover | A19-27 |
| MATH | Control | Red Clover | A28-35 |
| NWYK | LBM | White Clover | N1-7 |
| NWYK | LBM | Red Clover | N8-13 |
| NWYK | Control | White Clover | N14-22 |
| NWYK | Control | Red Clover | N23-31 |
| SHDV | LBM | White Clover | S1-8 |
| SHDV | LBM | Red Clover | S9-15 |
| SHDV | LBM | Black Medic | S17 |
| SHDV | Control | White Clover | S18-26 |
| SHDV | Control | Red Clover | S29-33 |
| SHDV | Control | Black Medic | S34-40 |
| TRBN | LBM | White Clover | T1-9 |
| TRBN | LBM | Red Clover | T10-18 |
| TRBN | LBM | Black Medic | T19 |
| TRBN | LBM | Lucerne | T20-23 |
| TRBN | Control | White Clover | T24-32 |
| TRBN | Control | Red Clover | T33-41 |
| WKNS | LBM | White Clover | W1-9 |
| WKNS | LBM | Red Clover | W10-18 |
| WKNS | LBM | Black Medic | W19-26 |
| WKNS | LBM | Lucerne | W27-29 |
| WKNS | Control | White Clover | W31-39 |
| WKNS | Control | Red Clover | W40-45 |
| WKNS | Control | Black Medic | W49-54 |
| WKNS | Control | Lucerne | W56-59 |
| WMPS | LBM | White Clover | M1-9 |
| WMPS | LBM | Red Clover | M10-18 |
| WMPS | LBM | Black Medic | M19-27 |
| WMPS | LBM | Lucerne | M28-37 |
| WMPS | Control | White Clover | M38-42 |
| WMPS | Control | White Clover | M41-42 |
| WMPS | Control | Red Clover | M43 |
| WMPS | Control | Lucerne | M48-49 |
| YATE | LBM | White Clover | Y1-9 |
| YATE | LBM | Red Clover | Y14-18 |
| YATE | LBM | Black Medic | Y19-26 |
| YATE | LBM | Lucerne | Y28-32 |
| YATE | Control | White Clover | Y33-42 |
| YATE | Control | Red Clover | Y43-50 |
| Inoculum |  | White Clover | I1-3 |
| Inoculum |  | Red Clover | I4-6 |
| Inoculum |  | Black Medic | I7-9 |
| Inoculum |  | Lucerne | I10-12 |
| RCR strain 221 |  | White Clover | R1-3 |
| RCR strain 221 |  | Red Clover | R4-6 |
| RCR strain 2011 |  | Black Medic | R7-9 |
| RCR strain 2011 |  | Lucerne | R10-12 |

**Table S2** Index of strains isolated from experimental plants. Site codes, Site treatment: LBM = site previously treated with commercial inoculum Control = site not treated with commercial inoculum.

| **Host legume** | **Rhizobia species** | **Bacterial strain code** |
| --- | --- | --- |
| White clover | *Rhizobium leguminosarum* sv. *trifolii* | RCR221* |
| Red clover |  |  |
| Alsike clover |  |  |
| Crimson clover |  | RCR226 |
| Black medic | *Ensifer meliloti* | RCR2011* |
| Lucerne |  |  |
| Sainfoin | *Rhizobium gallicum* sv. *gallicum* | RCR3007 |
| Birdsfoot trefoil | *Mesorhizobium loti* | RCR3002 |
| Large birdsfoot trefoil |  | RCR3209 |
| Meadow pea | *Rhizobium leguminosarum* sv. *viciae* | RCR1001* |

**Table S3** Rhizobia species and their UK host legumes, with details of strains contained in the Rothamsted Culture Collection. The ‘*’ indicates that the strain was contained in LegumeLINK commercial inoculum
